# A cell-permeable dominant-negative survivin protein induces apoptosis and sensitizes prostate cancer cells to TNF-α therapy

**DOI:** 10.1186/1475-2867-10-36

**Published:** 2010-10-01

**Authors:** Chun Hei Antonio Cheung, Xueying Sun, Jagat R Kanwar, Ji-Zhong Bai, LiTing Cheng, Geoffrey W Krissansen

**Affiliations:** 1 Department of Molecular Medicine & Pathology, Faculty of Medical and Health Sciences, The University of Auckland, Auckland, New Zealand; 2 The Hepatosplenic Surgery Center/Department of General Surgery, the First Clinical Medical School of Harbin Medical University, China; 3 Graduate Institute of Vaccine Technology, National Pingtung University of Science and Technology, Pingtung, Taiwan R.O.C; 4 National Institute of Cancer Research, National Health Research Institutes, Tainan 70456, Taiwan R.O.C; 5 Laboratory of Immunology and Molecular Biomedical Research (LIMBR), Centre for Biotechnology and Interdisciplinary Biosciences (BioDeakin), Institute for Technology & Research Innovation (ITRI), Deakin University, Geelong, Technology Precinct (GTP), Pigdons Road, Victoria 3217, Australia

## Abstract

**Background:**

Survivin is a member of the inhibitor-of-apoptosis (IAP) family which is widely expressed by many different cancers. Overexpression of survivin is associated with drug resistance in cancer cells, and reduced patient survival after chemotherapy and radiotherapy. Agents that antagonize the function of survivin hold promise for treating many forms of cancer. The purpose of this study was to investigate whether a cell-permeable dominant-negative survivin protein would demonstrate bioactivity against prostate and cervical cancer cells grown in three dimensional culture.

**Results:**

A dominant-negative survivin (C84A) protein fused to the cell penetrating peptide poly-arginine (R9) was expressed in *E. coli *and purified by affinity chromatography. Western blot analysis revealed that dNSurR9-C84A penetrated into 3D-cultured HeLa and DU145 cancer cells, and a cell viability assay revealed it induced cancer cell death. It increased the activities of caspase-9 and caspase-3, and rendered DU145 cells sensitive to TNF-α via by a mechanism involving activation of caspase-8.

**Conclusions:**

The results demonstrate that antagonism of survivin function triggers the apoptosis of prostate and cervical cancer cells grown in 3D culture. It renders cancer cells sensitive to the proapoptotic affects of TNF-α, suggesting that survivin blocks the extrinsic pathway of apoptosis. Combination of the biologically active dNSurR9-C84A protein or other survivin antagonists with TNF-α therapy warrants consideration as an approach to cancer therapy.

## Background

Survivin is a member of the inhibitors of apoptosis (IAP) family. Overexpression of survivin renders cancer cells resistant to anti-cancer therapy including chemotherapy and radiation therapy [1-5]. It causes oral cancer cells to be resistant to the anti-mitotic compounds vincristine and colchicine, such that down-regulation of survivin restores their drug sensitivity [2]. Overexpression of survivin inhibited the tamoxifen and cisplatin-induced apoptosis of human breast and gastric cancer cells [3,5]. It enhanced the repair of DNA double-strand breaks in radiation-treated oral cancer cells by upregulating the molecular sensor of DNA damage, Ku70 [4]. The level of survivin expression was inversely related to the degree of apoptosis, and positively related to the risk of local tumor recurrence in rectal cancer patients treated with radiotherapy [6]. Patients with gastric tumors that express low levels of survivin appear to have a longer mean survival time after cisplatin treatment than patients with high levels of expression [5]. Survivin expression is associated with the metastasis of human prostate cancer to bone [7]. Thus, survivin plays an important role in tumorigenesis and tumor metastasis, and where levels of survivin expression serve as an indicator of therapeutic effectiveness.

At the molecular level, survivin is bifunctional in that it is a suppressor of apoptosis and plays a central role in cell division. A study using surface plasmon resonance spectroscopy and immunoprecipitation analysis showed that a recombinant survivin protein was able to bind directly to both caspase-3 and caspase-7 with nanomolar affinity [8]. Targeting of survivin by siRNA induces the activation of caspase-9 and caspase-3 in various cancer cells [8,9]. It appears to be mitochondrial survivin rather than cytosolic survivin that inhibits apoptosis through interference with caspases [8,10,11]. Survivin also plays a role in inhibiting the caspase-independent apoptosis of cancer cells [12]. Translocation of the apoptosis-inducing factor (AIF) from the cytoplasm to the nucleus is a molecular indicator of the caspase-independent apoptosis of cells. Down-regulation of survivin by siRNA induces the translocation of AIF from the cytoplasm to the nucleus in various cancer cells [12].

Progress in the development of survivin inhibitors has been slow despite the fact that survivin plays multiple roles in cancer cell survival, and renders cancers insensitive to chemotherapy. In the past ten years only a few small molecule inhibitors of survivin have been developed and only one survivin inhibitor, YM155, has reached clinical trials [13-17]. Therefore, it is of interest to identify novel macromolecular inhibitors of survivin, and to explore their clinical utility. The 3D-structure of survivin has been determined by x-ray crystallography, which together with the gene sequence reveals that the 16.5 kDa survivin protein monomer comprises an N-terminal Zn^2+^-binding baculovirus IAP repeat (BIR) domain consisting of a three-stranded anti-parallel β-sheet surrounded by four small α helices that is linked to a 65 A° amphipathic C-terminal α-helix [18-20]. Survivin exists as a dimer and has an extensive dimerization interface along a hydrophobic surface on the BIR domain of each survivin monomer. Mutagenesis studies have shown that the BIR domain plays a key role in the anti-apoptotic function of survivin. Thus, point mutations such as C84A in the BIR domain prevent requisite dimerization of survivin, producing a dominant-negative mutant that interferes with the anti-apoptotic function of native survivin [21-23]. A Thr34 residue is located at the amino-terminal end of helix II of the BIR, surrounded by a sequence that matches the consensus phosphorylation site S/T-P-X-R for the mitotic kinase complex, p34cdc2-cyclin B1. Mutation of Thr34 to Ala (T34A) removes the phosphorylation site and prevents survivin from binding to activated caspase-9, creating a dominant-negative survivin molecule that disrupts cell division and induces apoptosis [24-26]. These and other dominant-negative forms of survivin are macromolecular inhibitors that have potential utility in the treatment of cancer.

Here we created a cell-permeable dominant-negative C84A survivin protein and investigated its biological activity against cancer cells grown in 3D culture.

## Results

### Production of a cell-permeable dominant-negative C84A survivin protein

**Figure 1:**
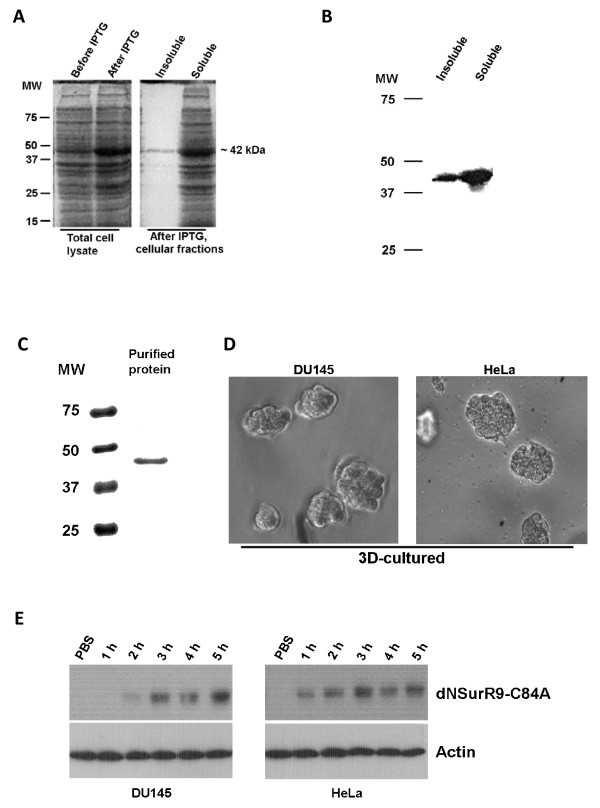
**Production of dNSurR9-C84A**. (**A**) SDS-PAGE analysis of proteins in bacteria transformed with pGEX-2T/dNSurR9-C84A. Bacteria were lysed before IPTG induction and 3 h after IPTG induction of protein expression. Analysis of the total lysate and insoluble and soluble fractions revealed a major ~42 kDa protein (position is indicated in the right-hand margin) induced by IPTG. Lane MW, molecular weights of protein markers in kDa. (**B**) Western blot analysis confirms the IPTG-induced ~42 kDa protein is dNSurR9-C84A. The insoluble and soluble fractions in (A) were Western blotted with a rabbit anti-survivin antibody, and immunoreactivity was detected with a peroxidase-conjugated goat anti-rabbit antibody. Lane MW, molecular weights of protein markers in kDa. (**C**) Purification of GST-tagged dNSurR9-C84A by affinity chromatography on glutathione agarose. The soluble fraction of lysates of IPTG-treated bacteria was chromatographed on glutathione agarose, and the glutathione eluted material subjected to SDS-PAGE and stained with Coomassie blue. (**D**) Formation of 3D cellular spheres of DU145 and HeLa cancer cells. Phase-contrast light microscopy of cellular spheres formed in semi-solid medium. (**E**) dNSurR9-C84A is able to penetrate into 3D-cultured DU145 and HeLa cells. Cells were incubated with dNSurR9-C84A for 1 to 5 h and cytoplasmic fractions Western blotted with an anti-GST antibody to detect GST-tagged dNSurR9-C84A. Blots were probed with an anti-actin antibody as a control.

A GST-tagged dominant-negative survivin protein (dNSurR9-C84A) was constructed based on the finding that mutation of Cys84 to Ala in the extreme C-terminal region of the BIR domain of survivin completely abrogates survivin's ability to inhibit apoptosis, transforming it into a dominant-negative inhibitor of survivin function [21]. The 5'-end of the cDNA sequence encoding dNSurR9-C84A was fused to a sequence encoding nine arginine residues (poly-arginine or R9 sequence) based on the finding that proteins fused with a poly-arginine carrier peptide are efficiently taken up by cells [27]. The pGEX-2T/dNSurR9-C84A construct encoding cell-permeable dNSurR9-C84A was transformed into BL21 *E.coli *cells and its expression induced with IPTG. SDS-PAGE analysis revealed an expressed protein of ~42 kDa in size that matched the expected molecular weight of recombinant dominant-negative survivin of 17 kDa fused to GST of 25 kDa (Figure 1A). Western blot analysis with an anti-survivin antibody confirmed that the ~42 kDa protein was GST-tagged dNSurR9-C84A, and that it was mostly expressed in the soluble fraction (Figure 1B). The dNSurR9-C84A protein was purified by glutathione affinity chromatography, giving a single product of 42 kDa (Figure 1C).

A preliminary study revealed that dNSurR9-C84A was able to penetrate into human DU145 prostate cancer cells in two dimensional (2D) cultures within 30 min of addition of the protein (Additional file 1) [28]. Here, the ability of dNSurR9-C84A to penetrate into 3D cultured cancer cells grown in semi-solid media containing Matrigel™was examined. DU145 prostate cancer and HeLa epithelial cervical cancer cells grown as 3D cultures (Figure 1D) were incubated with either PBS control or dNSurR9-C84A (20 μg/mL) for 1 to 5 h, and the cytosolic fraction of the cells was subjected to Western blotting. Cytosolic appearance of dNSurR9-C84A in DU145 and HeLa cells was observed as early as 1 to 2 h after addition of dNSurR9-C84A (Figure 1E).

### dNSurR9-C84A reduces the viability of DU145 and HeLa cancer cells

**Figure 2:**
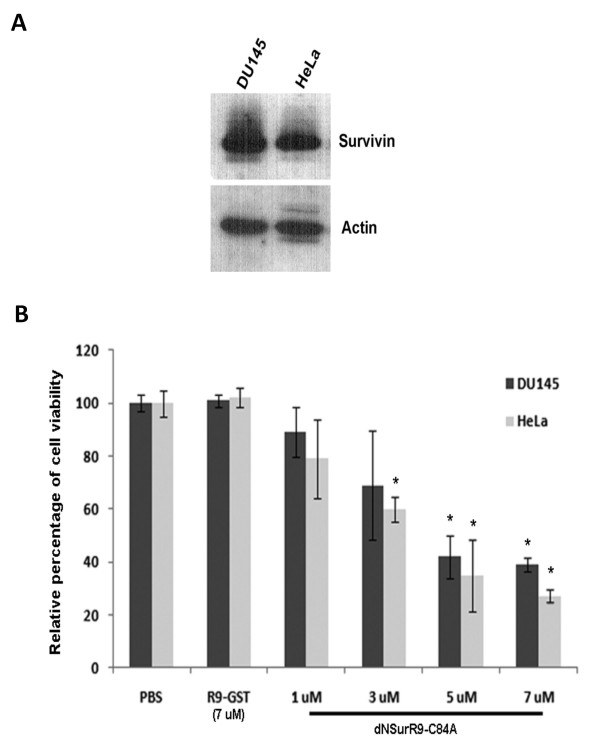
**dNSurR9-C84A induces cancer cell death**. (**A**) Total lysates of 3D-cultured DU145 and HeLa cells were Western blotted with an anti-survivin antibody which indicated endogenous expression of survivin. Blots were probed with an anti-actin antibody as a control. (**B**) dNSurR9-C84A induces cancer cell death in a concentration-dependent manner. 3D cultures of DU145 and HeLa cells were treated with dNSurR9-C84A for 36 h and cell viability measured by MTT assay. A statistically significant difference in the viability of cells treated with dNSurR9-C84A versus R9-GST is denoted by "*". *p < 0.05.

The effect of dNSurR9-C84A on the viability of 3D cultures of DU145 and HeLa cells, which endogenously express survivin as assessed by Western blot analysis (Figure 2A), was examined. A preliminary study revealed that treatment of 2D-cultured DU145 and HeLa cancer cells with dNSurR9-C84A for 24 h induced caspase-3-associated apoptosis, whereas similar treatment did not effect survivin-independent human umbilical vein endothelial cells (HUVEC) (Additional file 2) [28]. DU145 and HeLa cells grown in 3D cultures were incubated for 36 h with 16, 48, 80 and 112 μg/mL (ie 1, 3, 5, and 7 μM, respectively) of dNSurR9-C84A or with a recombinant R9-tagged GST protein (7 μM) which served as a negative control. dNSurR9-C84A at 7 μM significantly (p < 0.01) reduced the cell viability of DU145 and HeLa cancer cells by 60% and 70%, respectively (Figure 2B). In contrast, R9-GST at 7 μM had no affect on cell viability (Figure 2B). Thus, dNSurR9-C84A is active in inducing the death of survivin-dependent cancer cells grown in 3D culture.

### dNSurR9-C84A increases the activity of caspases-3 and 9 in a time-dependent fashion

**Figure 3:**
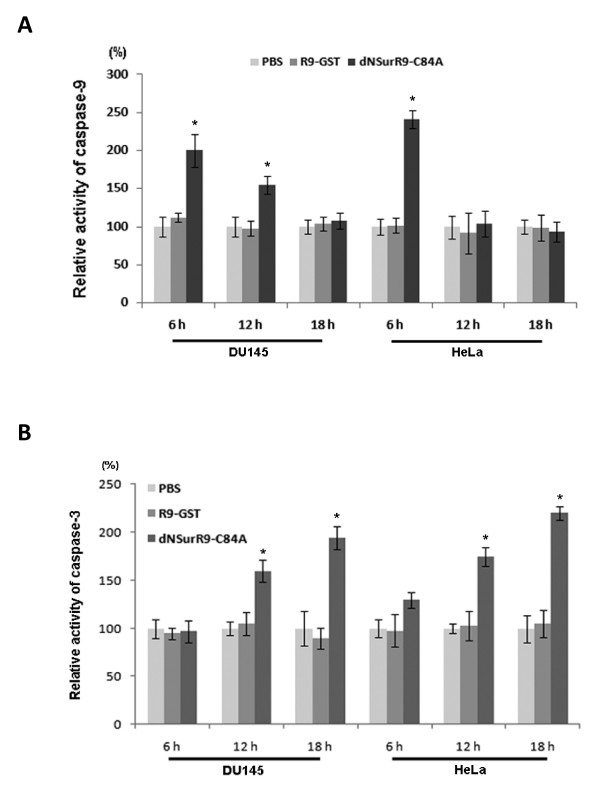
**dNSurR9-C84A upregulates the activity of caspase-9 and caspase-3 in cancer cells**. (**A, B**) 3D cultures of DU145 and HeLa cells were treated with dNSurR9-C84A, R9-GST and PBS, and the activities of caspase-9 (A) and caspase-3 (B) were measured 6, 12 and 18 h post-treatment. A statistically significant difference in caspase activity between cells treated with dNSurR9-C84A and R9-GST is denoted as "*". *p < 0.05.

Targeting of survivin expression has previously been shown to induce cancer cell death through activation of intrinsic apoptosis mediated by caspases-3 and 9 [10,29,30]. In order to determine whether dNSurR9-C84A decreases cell viability via activation of caspases, the activities of caspase-3 and 9 were measured in DU145 and HeLa cancer cells treated with 5 μM of dNSurR9-C84A. dNSurR9-C84A increased the activity of caspases-3 and 9 by 100 to 150% (p < 0.05) in both cancer cell lines, compared to control treated cells (Figure 3A, B). The activity of caspase-9 was induced as early as 6 h post-treatment in DU145 and HeLa cells, and returned to background levels at 18 h post-treatment (Figure 3A). The induction of caspase-3 activity was delayed compared to that of caspase-9. Increased caspase-3 activity was seen in HeLa cells at 6 h post-treatment, but not until 12 h post-treatment in the case of DU145 cells (Figure 3B). Further, the activity of caspase-3 steadily increased with time in both cell lines reaching a peak at 18 h post-treatment. In contrast, treatment of the cells with R9-GST did not lead to an increase in the activity of the caspases compared to the PBS control.

### dNSurR9-C84A restores cell sensitivity to TNF-α via activation of caspase-8

**Figure 4:**
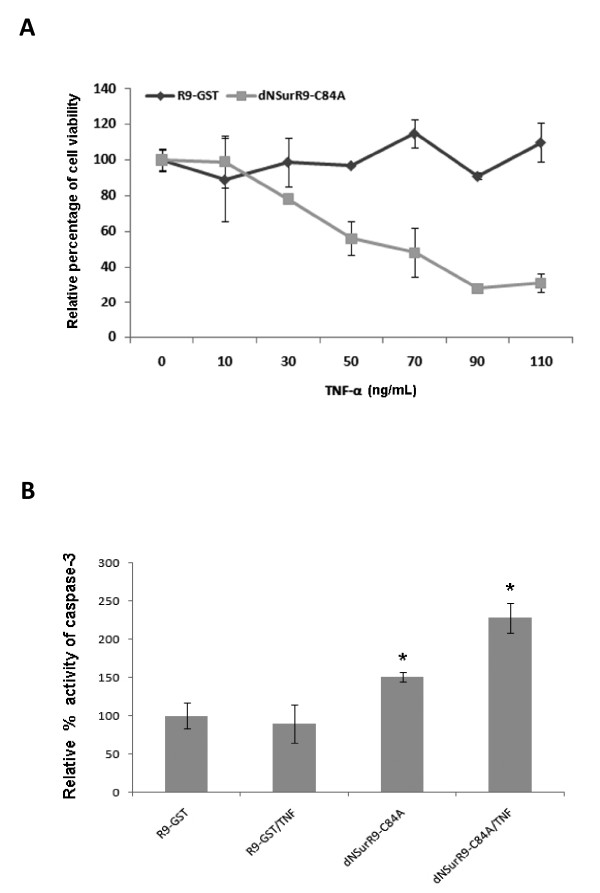
**dNSurR9-C84A renders DU145 cancer cells susceptible to the proapoptotic effects of TNF-α**. (**A**) 3D cultures of DU145 cells were treated with either R9-GST or dNSurR9-C84A in combination with increasing concentrations of TNF-α for 36 h, and cell viability measured by the MTT assay. (**B**) 3D cultures of DU145 cells were treated with either R9-GST or dNSurR9-C84A alone or in combination with TNF-α for 18 h and the activity of caspase-3 was measured. A statistically significant difference versus R9-GST is denoted as "*". *p < 0.05.

Here we investigated the possibility that dNSurR9-C84A might activate the caspase-8-mediated pathway of extrinsic apoptosis, and thereby augment the efficacy of TNF-α treatment of cancer. DU145 is an androgen-independent prostate cancer cell line that displays resistance to TNF-α treatment [31]. As would be expected, combinational treatment of DU145 cells with R9-GST and TNF-α (10 to 110 ng/mL) for 36 h had no affect on cell viability (Figure 4A). In contrast, treatment with the combination of 3 μM dNSurR9-C84A and TNF-α (50 to 110 ng/ml) caused a marked reduction in cell viability (Figure 4A). The reduction in cell viability was associated with significant 130, 90 and 140% increases in caspase-3 activity, compared to treatment with R9-GST (p < 0.05), dNSurR9-C84A (p < 0.05), and R9-GST in combination with TNF-α (p < 0.05), respectively (Figure 4B). These results demonstrate that antagonism of survivin renders DU145 prostate cancer cells sensitive to the proapoptotic effects of TNF-α.

**Figure 5:**
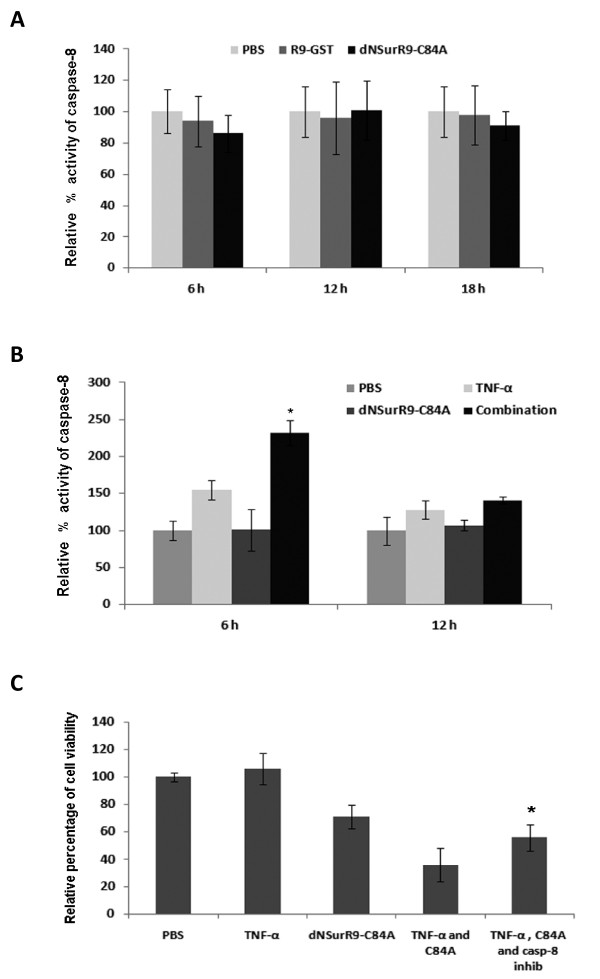
**dNSurR9-C84A in combination with TNF-α upregulates the expression of caspase-8 activity**. (**A**) dNSurR9-C84A does not induce caspase-8 activity in 3D cultured DU145 cells. Cells were treated with PBS, R9-GST and dNSurR9-C84A for 6 to 12 h and the activity of caspase-8 measured. (B) dNSurR9-C84A potentiates the upregulation of caspase-8 in response to TNF-α. 3D-cultures of cells were treated with PBS, TNF-α, dNSurR9-C84A and the combination of dNSurR9-C84A and TNF-α for 6 and 12 h. The activity of caspase-8 was measured. A statistically significant difference in the caspase-8 activity of cells treated with the combination of dNSurR9-C84A and TNF-α versus TNF-α alone is denoted as "*". *p < 0.05. (**C**) 3D-cultures of DU145 cells were treated with PBS, TNF-α, dNSurR9-C84A, and the combination of dNSurR9-C84A and TNF-α in the presence or absence of the caspase-8 specific inhibitor Z-IETD-FMK for 36 h. Cell viability was assessed by the MTT assay. A statistically significant difference in the caspase-8 activity of cells treated with the combination of dNSurR9-C84A, TNF-α, and Z-IETD-FMK versus the combination of dNSurR9-C84A and TNF-α is denoted as "*".*p < 0.05.

TNF-α induces cell apoptosis through the activation of caspase-8 which leads to the subsequent activation of caspase-3 [32]. Down-regulation of survivin by siRNA does not induce the activation of caspase-8 in cancer cells [33]. Here we asked whether the combinational treatment of 3D-cultured DU145 cells with dNSurR9-C84A and TNF-α leads to activation of caspase-8. As expected, treatment of DU145 cells with 3 μM dNSurR9-C84A did not increase the activity of caspase-8, compared to cells treated with R9-GST or PBS (Figure 5A). In contrast, treatment of cells with the combination of 50 ng/mL of TNF-α and 3 μM dNSurR9-C84A significantly increased caspase-8 activity at 6 h post-treatment by 125 (p < 0.01), 75 (p < 0.05), and 124 (p < 0.05)%, compared to treatment with PBS, TNF-α, and dNSurR9-C84A, respectively (Figure 5B). The increased caspase-8 activity returned to background levels by 12 h post-treatment. Inclusion of 5 μM of the caspase-8 inhibitor Z-IETD-FMK significantly (p < 0.05) inhibited the decrease in cell viability of DU145 cells caused by treatment with the combination of TNF-α and dNSurR9-C84A (Figure 5C).

## Discussion

The protein expression profiles of 2D and 3D-cultured cancer cells are different [34]. In comparison to 2D-cultured cells, the behavior of cells cultured under 3D conditions is more reminiscent of that of cells growing *in situ *[34]. Hence 3D cell cultures represent an improved *in vitro *system to model the behavior of potential therapeutic drugs. Here we used a 3D cell culture system to test the ability of the survivin antagonist dNSurR9-C84A to kill prostate and cervical cancer cells. This is the first report of the production and characterization of a "cell-permeable" recombinant form of survivin in which cysteine at position 84 in the zinc-coordination site in the BIR domain has been substituted with alanine. The architecture of the BIR domain is disturbed by mutation of the C84 residue to alanine, and the ability of survivin to dimerize and interfere with caspases is inhibited [21-23]. Herein, we revealed that dNSurR9-C84A was able to kill 3D-cultured prostate and cervical cancer cells, where killing was associated with increased levels of caspase-3, and with DNA fragmentation in the case of DU145 cells. Further, dNSurR9-C84A rendered prostate cancer cells sensitive to the proapoptotic effects of TNF-α by potentiating the upregulation of caspase-8 activity, suggesting that survivin inhibits the extrinsic pathway of apoptosis. In accord, melatonin has been shown to reduce both survivin and Bcl-2 protein levels and subsequently increase the sensitivity of human PC3 prostate cancer cells to TNF-α [35]. Further, adhesion of the aggressive prostate cancer cell line PC3 to fibronectin results in upregulation of survivin and protects the cells from apoptosis induced by TNF-α [36]. Adenoviral expression of dominant negative T34A survivin counteracted the ability of fibronectin to protect the cells from undergoing apoptosis, whereas wild-type survivin protected non-adherent cells from TNF-α-induced apoptosis.

It remains to be determined whether survivin inhibits TNF-α signaling through direct interaction with caspase-8 or via an indirect interaction, but at least one study indicates a direct interaction is unlikely [10]. Survivin is believed to be primarily involved in the intrinsic apoptosis pathway. Biochemical and structural analysis revealed that survivin physically binds to caspase-3 and caspase-7 and inhibits their activities [8,10]. Therefore, it is possible that survivin interferes with the TNF-α simulated apoptosis through both indirect regulation of the caspase-8 activity and direct regulation of caspase-3 activity.

Our laboratory has previously shown that intratumoral delivery of a plasmid expressing dNSur-C84A causes tumour apoptosis in an animal model of lymphoma [22]. However, gene therapy clinical trials are hindered by immune responses that can lead to overwhelming inflammation and the death of patients [37]. It is pertinent to devise other treatment forms where gene therapy proves to be problematic. Macromolecular protein approaches targeting tumour survival factors, as exemplified here with dNSurR9-C84A may hold promise for therapy.

## Conclusions

In conclusion, a cell-permeable form of the dominant-negative (C84A) survivin protein has been successfully produced, and demonstrated to exert biological activity in being able to kill prostate and cervical cancer cells. Importantly, it was discovered that dNSurR9-C84A antagonizes survivin's ability to suppress TNF-α signaling, rendering prostate cancer cells susceptible to the proapoptotic effects of TNF-α. dNSurR9-C84A is a both a novel tool for probing the function of survivin, and a potential therapeutic agent for augmenting cancer therapy.

## Materials and methods

### Cell Lines, antibodies and reagents

The cell lines DU145 (human prostate carcinoma), and HeLa (cervical epithelial carcinoma) were purchased from the American Type Culture Collection (ATCC, Manassas, VA). DU145 cells were cultured in a mixture of Matrigel™(BD Biosciences, San Jose, CA) and RPMI-1640 (Gibco, Grand Island, NY) in a 1:1 ratio. HeLa cells were cultured in a mixture of Matrigel and Dulbecco's modified Eagle medium (DMEM) (Gibco, Grand Island, NY) in a 1:1 ratio. Both the latter media were supplemented with 10% foetal bovine serum, penicillin (100 U/mL), streptomycin (100 μg/mL) and L-glutamine (0.29 mg/mL). Cells were cultured in the semi-solid medium for five days to allow the formation of three dimensional cellular spheres. Antibodies used in this study included a goat anti-GST antibody (Amersham Biosciences, Freiburg, Germany) and a rabbit anti-human/mouse survivin antibody (Alpha Diagnostic, San Antonio, TX).

### Construction of a dominant-negative survivin expression vector

A dominant-negative cell-permeable form of survivin (dNSurR9-C84A) comprising 9 N-terminal arginine residues (R9, cell-permeable peptide carrier) fused to the C84A dominant-negative survivin mutant was constructed for the study. Briefly, the cDNA of a pcDNA3 expression plasmid encoding dominant-negative survivin which contains the entire coding region of mouse survivin (nucleotides 75-583; GenBank accession No. NM_009689) and a T-to-G substitution at nucleotide 354 that changes the cysteine residue at amino acid 84 in the extreme C-terminal region of the BIR domain to an alanine was amplified using the sense primer 5'- GGGGATCCATGCGACGACGACGACGACGACGACGACGAGGAGCTCCGGCGCTGCCCCAG-3' (encodes R9) and the antisense primer 5'-GGGATCCTTAGGCAGCCAGC-3'[22]. The resulting PCR product was subcloned into pGEM-T (Promega Corp., Madison, WI), excised by digestion with *BamH*I, and cloned into the expression vector pGEX-2T (Pharmacia Biotech, Piscataway, NJ) to give the vector pGEX-2T/dNSurR9-C84A. The integrity of the pGEX-2T/dNSurR9-C84A vector was confirmed by DNA sequence analysis.

### Production of a recombinant dominant-negative survivin protein dNSurR9-C84A

The dNSurR9-C84A expression vector was transformed into DH5α bacteria, and the transformants were cultured at 37°C in LB medium containing 100 mg/L of ampicillin. When the OD_600 nm _reached 0.7, protein expression was induced with IPTG (0.7 mM) (Invitrogen, Carlsbad, CA) at 30°C for 3 h. The bacteria were pelleted and lysed in STE buffer containing 0.1 mg/ml of lysozyme, 10 mM dithiothreitol and 0.7% sarkosyl, and sonicated for 30 sec. The GST-tagged recombinant dNSurR9-C84A protein was purified by glutathione-Sepharose chromatography, and dialyzed twice against PBS.

### SDS-PAGE and Western blotting

Cells were lysed with lysis buffer (10 mM Tris, 1 mM EDTA, 1 mM DTT, 60 mM KCl, 0.5% NP-40 and Complete Protease Inhibitor Cocktail Tablet from Roche, Germany, containing a mixture of protease inhibitors), and proteins were resolved on 12% polyacrylamide SDS gels under reducing conditions. The gels were either stained with Coomassie blue or proteins electrophoretically transferred to Hybond C Extra nitrocellulose membranes (Amersham Life Science, Amersham, UK). The membranes were blocked overnight at 4°C with 5% non-fat milk powder, incubated with primary antibodies for 90 min at RT, and then with a horseradish peroxidase-conjugated secondary antibody (Sigma Chemical Co., St Louis, MD). Immunoreactivity was detected by Enhanced Chemiluminescence (ECL) (Amersham International, Buckingham, UK) and autoradiography.

### Cell viability assays

Cells (4 × 10^3^) were cultured in a semi-solid culture medium comprised of Matrigel™and RPMI for five days to allow the formation of three dimensional cellular spheres. The cellular spheres were treated with test reagents for 36 h. Dispase (BD Biosciences, San Jose, CA), a bacillus-derived neutral metalloprotease, was used to recover cells cultured in the Matrigel™. Live cells were resuspended in PBS and cell viability was analyzed with the CellTiter96 MTS cell proliferation assay kit (Promega Corp., Madison, WI) with measurements being made on a 96-well plate reader (Bio-Tek Instruments Inc.).

### Measurement of caspase activity

The activity of caspase-3 in cell lysates was determined with the Apo-ONE^® ^Homogeneous Caspase-3/-7 apoptosis detection kit (Promega Crop., Madison, WI). Caspase-8 activity was determined with the Caspase-8 Fluorometric Protease Assay Kit (Biovision, Mountain View, CA). Caspase-9 activity was determined with the Caspase-9 Fluorometric Protease Assay Kit (Biovision, Mountain View, CA).

### Measurement of DNA fragmentation

Cells were stained with the TUNEL reagent (*In-Situ *Apoptosis Detection kit, Roche Diagnostic, Mannheim, Germany) to detect DNA fragmentation, counter-stained with propidium iodide (PI), and examined by fluorescence microscopy.

### Statistical analysis

The Student's *t*-test was used with p < 0.05 indicating a statistically significant difference.

## Abbreviations

BIR: baculovirus IAP repeat; dNSurR9-C84A: poly-arginine tagged dominant-negative survivin protein; DAPI: 4',6-diamidino-2-phenylindole; IAP: inhibitor of apoptosis protein; IPTG: isopropyl β-D-1-thiogalactopyranoside; TUNEL: transferase (TdT)-mediated dUTP-biotin nick end labelling.

## Competing interests

The authors declare that they have no competing interests.

## Authors' contributions

CHAC performed all the experiments and drafted the manuscript. XS and JK cosupervised the student. JB contributed to analyses involving confocal microscopy. LC contributed to the purification of the recombinant protein. GWK was the Principle Investigator who directed the work, supervised the student, and revised the manuscript for publication. All authors read and approved the final manuscript.

## Supplementary Material

Additional file 1 **dNSurR9-C84A is rapidly taken up by DU145 cells**. DU145 cells were incubated with either PBS (control) or dNSurR9-C84A protein for 30 min. Cells were stained with an anti-GST antibody and counter-stained with DAPI. Cells were examined by standard immunofluorescence microscopy (top 2 rows) or by confocal microscopy (bottom row). Green fluorescence indicates the presence of GST-tagged dNSurR9-C84A in the cytoplasm. The cells were photographed, and the images merged (right-hand panels). Click here for file

Additional file 2 **dNSurR9-C84A inhibits the viability of DU145 and HeLa cells**. (**A**) Cells were incubated with either PBS (control) or increasing concentrations of dNSurR9-C84A for 12 h, and cell viability was assessed. A statistically significant difference in the viability between cells treated with dNSurR9-C84A and PBS is denoted as "*". *p < 0.05. (**B**) dNSurR9-C84A induces caspase-3/-7 activities in DU145 cells in a dose-dependent fashion. DU145 cells were incubated with increasing concentrations of dNSurR9-C84A for 90 min. The cells were lysed and caspase-3/-7 activity in the cell lysate measured. A statistically significant difference in the caspase activity between cells treated with dNSurR9-C84A and PBS was denoted as "*". *p < 0.05. (**C**) dNSurR9-C84A induces DNA fragmentation in DU145 cells. Cells were incubated with PBS or dNSurR9-C84A for 3 h, and stained (green) with the TUNEL agent. The permeabilized cells were counter-stained (red) with propidium iodide. Click here for file
