# Correction: A cell-permeable dominant-negative survivin protein induces apoptosis and sensitizes prostate cancer cells to TNF-α therapy

**DOI:** 10.1186/1475-2867-10-43

**Published:** 2010-10-28

**Authors:** Chun Hei Antonio Cheung, Xueying Sun, Jagat R Kanwar, Ji-Zhong Bai, LiTing Cheng, Geoffrey W Krissansen

**Affiliations:** 1 Department of Molecular Medicine & Pathology, Faculty of Medical and Health Sciences, The University of Auckland, Auckland, New Zealand; 2 Alternate address: The Hepatosplenic Surgery Center/Department of General Surgery, the First Clinical Medical School of Harbin Medical University, China; 3 Graduate Institute of Vaccine Technology, National Pingtung University of Science and Technology, Pingtung, Taiwan; 4 National Institute of Cancer Research, National Health Research Institutes, Tainan 70456, Taiwan; 5 Laboratory of Immunology and Molecular Biomedical Research (LIMBR), Centre for Biotechnology and Interdisciplinary Biosciences (BioDeakin), Institute for Technology & Research Innovation (ITRI), Deakin University, Geelong, Technology Precinct (GTP), Pigdons Road, Victoria 3217, Australia

## Correction

Since publication of our article [1], we have regrettably noticed an error. The molarities of 16, 48, 80 and 112 μg/mL solutions of dNSurR9-C84A of molecular weight 42 kDa were incorrectly given as 1, 3, 5, and 7 μM. The correct molarities which should be used throughout are 0.38, 1.1, 1.9, and 2.7 μM, respectively.
