# Supplementary figures and images for: A cell-permeable dominant-negative survivin protein induces apoptosis and sensitizes prostate cancer cells to TNF-α therapy
Source: Cancer Cell Int. 2010 Oct 1;10:36. doi: 10.1186/1475-2867-10-36 (PMC2958862; doi:10.1186/1475-2867-10-36)

## Slide 1
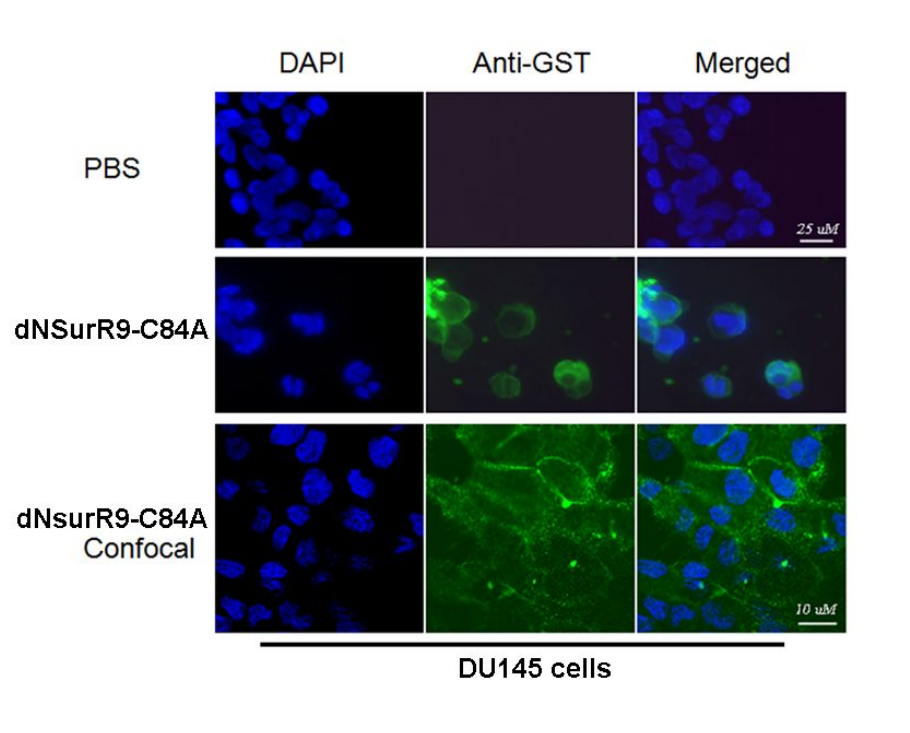

Supplement: Additional file 1 — dNSurR9-C84A is rapidly taken up by DU145 cells. DU145 cells were incubated with either PBS (control) or dNSurR9-C84A protein for 30 min. Cells were stained with an anti-GST antibody and counter-stained with DAPI. Cells were examined by standard immunofluorescence microscopy (top 2 rows) or by confocal microscopy (bottom row). Green fluorescence indicates the presence of GST-tagged dNSurR9-C84A in the cytoplasm. The cells were photographed, and the images merged (right-hand panels). [file 1475-2867-10-36-S1.PPT]
