# Supplementary material for: A cell-permeable dominant-negative survivin protein induces apoptosis and sensitizes prostate cancer cells to TNF-α therapy
Source: Cancer Cell Int. 2010 Oct 1;10:36. doi: 10.1186/1475-2867-10-36 (PMC2958862; doi:10.1186/1475-2867-10-36)
Supplement: Additional file 2 — dNSurR9-C84A inhibits the viability of DU145 and HeLa cells. (A) Cells were incubated with either PBS (control) or increasing concentrations of dNSurR9-C84A for 12 h, and cell viability was assessed. A statistically significant difference in the viability between cells treated with dNSurR9-C84A and PBS is denoted as "*". *p < 0.05. (B) dNSurR9-C84A induces caspase-3/-7 activities in DU145 cells in a dose-dependent fashion. DU145 cells were incubated with increasing concentrations of dNSurR9-C84A for 90 min. The cells were lysed and caspase-3/-7 activity in the cell lysate measured. A statistically significant difference in the caspase activity between cells treated with dNSurR9-C84A and PBS was denoted as "*". *p < 0.05. (C) dNSurR9-C84A induces DNA fragmentation in DU145 cells. Cells were incubated with PBS or dNSurR9-C84A for 3 h, and stained (green) with the TUNEL agent. The permeabilized cells were counter-stained (red) with propidium iodide. [file 1475-2867-10-36-S2.PPT]

## Slide 1
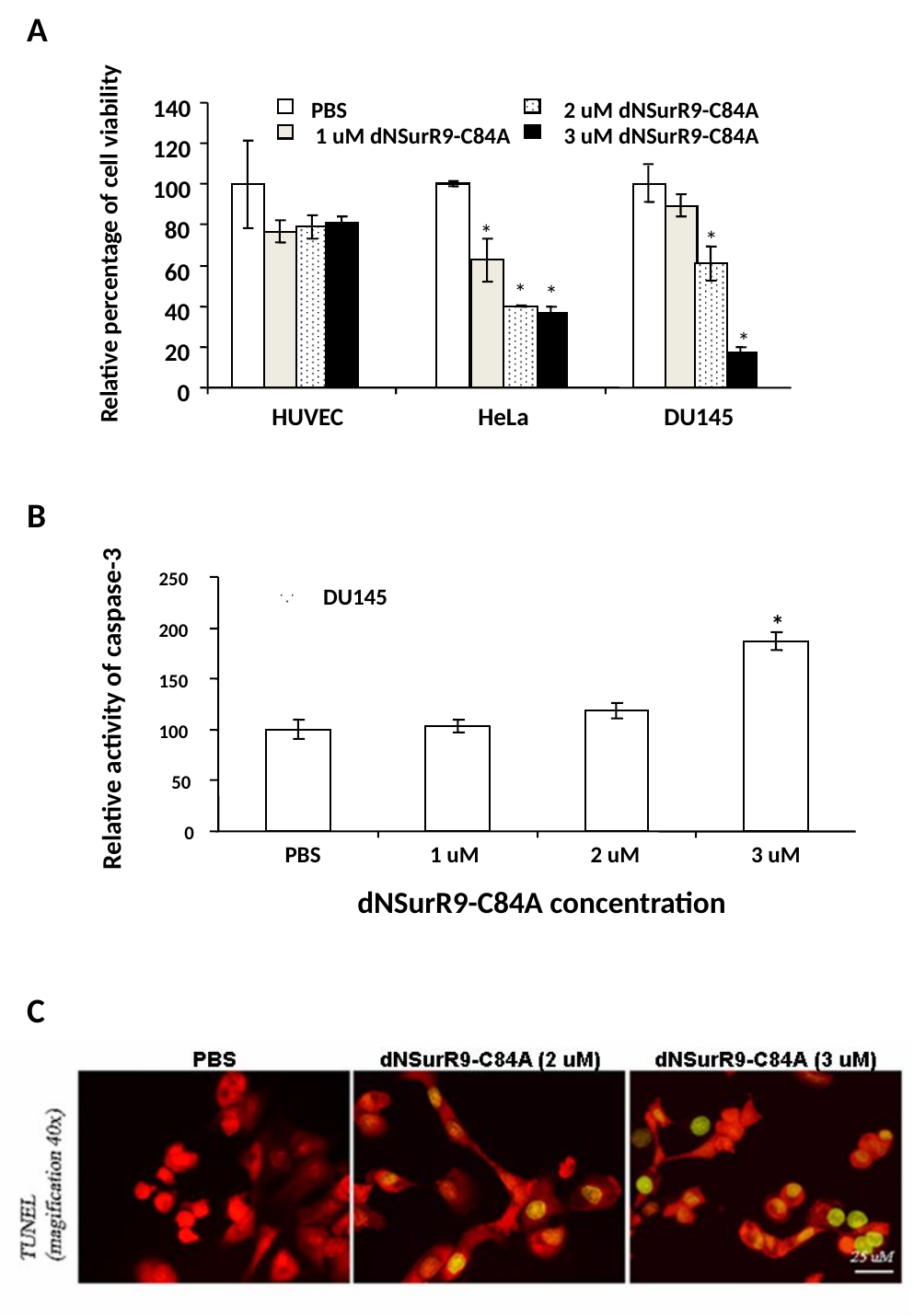

A
140
PBS
2 uM dNSurR9-C84A
 1 uM dNSurR9-C84A
3 uM dNSurR9-C84A
120
100
80
 *
 *
Relative percentage of cell viability
60
 *
 *
40
 *
20
0
HUVEC
HeLa
DU145
B
250
 *
200
150
Relative activity of caspase-3
100
50
0
PBS
1 uM
2 uM
3 uM
dNSurR9-C84A concentration
DU145
C
